# MicroRNA-665 facilitates cell proliferation and represses apoptosis through modulating Wnt5a/β-Catenin and Caspase-3 signaling pathways by targeting TRIM8 in LUSC

**DOI:** 10.1186/s12935-021-01913-z

**Published:** 2021-04-15

**Authors:** Tian-Jun Chen, Qi Zheng, Fei Gao, Tian Yang, Hui Ren, Yang Li, Ming-Wei Chen

**Affiliations:** 1 Respiratory Department, The First Affiliated Hospital, Xi’an Jiaotong University, Xi’an, 710061 People’s Republic of China; 2 First Department of Medical Oncology, Affiliated Shaanxi Provincial Cancer Hospital, College of Medicine, Xi’an Jiaotong University, Xi’an, 710061 Shaanxi China; 3 Hua-Shan Central Hospital of Xi’an, Xi’an, 710043 People’s Republic of China

**Keywords:** MiR-665, TRIM8, Lung squamous cell carcinoma, Proliferation, Apoptosis

## Abstract

**Background:**

MicroRNAs (miRNAs) are involved in the oncogenesis, development and transformation of lung squamous cell carcinoma (LUSC). miR-665 is clinically significant and acts as a pivotal function in some cancers. Nevertheless, the effects and the potential mechanisms of miR-665 in human LUSC are still unknown.

**Methods:**

To analyse the clinical significant of miR-665 in human LUSC, quantitative real-time PCR (qRT-PCR) was use to measure miR-665 expression in LUSC specimen tissues and cell lines. Tripartite motif 8 (TRIM8) was verified a target of miR-665 by performing bioinformatic prediction and luciferase reporter assay. The expression levels of TRIM8 were examined through qRT-PCR and Western blotting in LUSC specimen tissues. CCK8 assay was fulfilled for analyzing the function in LUSC cell proliferation. Flow cytometry was used to detect cell and apoptosis. TRIM8 silencing and overexpression further verified the biological effects as those caused by miR-665.

**Results:**

Here we reported that miR-665 expression was upregulated in LUSC specimen tissues and cell lines. High miR-665 levels were related to differentiation, tumor size and TNM stage. miR-665 mimics facilitated LUSC cell growth and cell cycle G1-S transition and repressed apoptosis. miR-665 inhibitor suppressed cell proliferation and G1-S transition and promoted apoptosis. miR-665 expression was negatively correlated with TRIM8 mRNA expression in LUSC. Luciferase reporter assay confirmed that TRIM8 was a direct target gene of miR-665. miR-665 mimics downregulated the TRIM8 levels, and miR-665 inhibitor upregulated the TRIM8 levels in LUSC cells. Particularly, silencing TRIM8 led to the similar effects of miR-665 mimics in LUSC cells. Overexpression of TRIM8 inhibited LUSC cell proliferation in vitro and in vivo. Furthermore, miR-665 promoted LUSC cell proliferation through facilitating the Wnt5a/β-catenin signaling pathway and restrained apoptosis via inhibiting Caspase-3 signaling pathway, whereas TRIM8 suppressed cell growth by repressing the Wnt5a/β-catenin signaling pathway and induced apoptosis through activating Caspase-3 signaling pathway.

**Conclusions:**

The current study demonstrates that miR-665 facilitates LUSC cell proliferation and cell cycle transition by regulation of the Wnt5a/β-Catenin signaling pathway and represses cell apoptosis via modulation of Caspase-3 signaling pathway by directly targeting TRIM8. These findings suggest that miR-665 might be a potential new target for LUSC therapy.

**Supplementary Information:**

The online version contains supplementary material available at 10.1186/s12935-021-01913-z.

## Background

Lung cancer is one of the most common causes of cancer-related death with increasing occurrence and mortality, accounting for more than 1.7 million deaths yearly worldwide [1]. In China, there are approximately 730,000 newly diagnosed and 610,000 deaths due to lung cancer in 2015 [2]. The proportion of non-small cell lung cancer (NSCLC) is about 85% in lung cancer cases. According to the histological and pathological features, NSCLC are classified as lung adenocarcinoma, lung squamous cell carcinoma (LUSC), lung neuroendocrine cancer and lung large cell carcinoma [3, 4]. LUSC ranks second in NSCLC and accounts for more than 30% of NSCLC patients [5]. Most patients with LUSC are diagnosed at an advanced stage and have a low 5-year survival rate [6]. Hence, it is importance to further discover the potential molecular mechanism underlying the oncogenesis and development of LUSC for improving the diagnosis, prevention and therapy.

MicroRNAs (miRNAs), a class of endogenous single strand RNA molecules (18–24 nt), regulate about a third of human gene expressions by targeting the 3′-untranslated regions (3′-UTR) of specific messenger RNAs [7, 8]. Previous studies reveal that miRNAs play crucial roles in a variety of cellular processes, such as cell proliferation, survival, cycle, apoptosis, migration, invasion, differentiation and drug sensitivity of tumors [9–12]. Moreover, miRNAs have been reported to be implicated oncogenesis and development [13]. LUSC carcinogenesis is a multifactorial and multistep biological process. Some studies have found that miRNAs are involved in the oncogenesis, development and transformation of LUSC, such as miR-182-5p, miR-198-5p, miR-223-3p, miR-372-3p [14–17]. Previous studies have shown that miR-665 is clinically significant and regulates oncogenesis and development in breast cancer, ovarian cancer, liver cancer, gastric cancer, pancreatic cancer, retinoblastoma and lymphoblastic leukemia [18–21]. However, the clinical significance, precise molecular mechanisms and biological functions of miR-665 in LUSC remain unclear. Recently, we discover that miR-665 is frequently upregulated in LUSC tissues and cell lines. We predict that miR-665 might target tripartite motif 8 (TRIM8) by using bioinformatics data. TRIM8 belongs to a member of the tripartite-motif (TRIM) protein family involved in all kinds of biological processes, including oncogenesis and development [22, 23]. Up to now, the function and mechanism of TRIM8 in plenty of cancers, including LUSC, is still unknown.

In the present study, we explored the roles and underlying mechanism of miR-665 in human LUSC. Our results showed that the miR-665 expressions were significantly upregulated in human LUSC specimen tissues and cell lines. miR-665 remarkably facilitated LUSC NCI-H226/SK-MES-1 cell proliferation and cell cycle transition through regulation of the Wnt5a/β-Catenin signaling pathway and repressed cell apoptosis via modulation of Caspase-3 signaling pathway by targeting TRIM8. TRIM8 silencing and overexpression further verified the biological effects as those caused by miR-665. These findings illustrated an oncogenic function of miR-665 in human LUSC, indicating that miR-665 might be a potential new target for LUSC therapy.

## Materials and methods

### Specimens from patients with LUSC

LUSC tumor tissues and paired adjacent non-tumor tissues were obtained from 69 patients at the Department of Thoracic Surgery, the First Affiliated Hospital of Medical College, Xi’an Jiaotong University, Xi’an, Shaanxi Province, China, between March 2015 and November 2018. All patients weren’t treated with chemotherapy or radiotherapy. We acquired informed consent from the patients before clinical tissue gathering. The clinicopathological parameters of patients were obtained by reviewing their pathology records. The clinical specimens were promptly preserved at − 80 °C after collection. The study was authorized by the Ethics Committee of The First Affiliated Hospital of Xi’an Jiaotong University.

### Animals

Six-week-old male BALB/c nude mice which were purchased from Guangdong Medical Laboratory Animal Center were fed under aseptic conditions. The animal experiments were fulfilled in accordance with the guidelines of the use of laboratory animals, and were authorized by the Institutional Animal Care and Use Committee of The First Affiliated Hospital of Xi’an Jiaotong University.

### Cell culture

Human LUSC cell lines NCI-H226, NCI-H1703, SK-MES-1 and NCI-H520, and normal human lung epithelial cells BEAS-2B were purchased from the Cell Bank (Shanghai Genechem Co., Ltd, China). All cell lines have been verified and haven’t been infected by other microbe. These cells were cultivated in RPMI-1640 (Gibco BRL, Grand Island, NY, USA), including 20 μg penicillin/mL, 20 μg streptomycin/mL, and 10% (v/v) fetal bovine serum (Gibco BRL, Grand Island, NY, USA) at 37 °C in the humidified incubator comprising 5% carbon dioxide.

### miR-665 mimics/inhibitor, TRIM8 siRNA synthesis and transfection

miR-665 mimics, miR-665 mimics negative control (Control, scrambled invalid RNA sequence), miR-665 inhibitor and negative control (NC, scrambled invalid RNA sequence) were designed and synthesized by GenePharma Corporation (Shanghai, China). After culturing NCI-H226/SK-MES-1 cells for 24 h in plates, the RNA oligonucleotides were transiently transfected into these cells with Lipofectamine TM-2000 (Invitrogen, Carlsbad, CA, USA), respectively. Small interfering RNAs (siRNAs) were designed for silencing TRIM8 gene expression. TRIM8 siRNA and negative control siRNA (NC-siRNA, scrambled invalid siRNA sequence) were synthesized by GenePharma Corporation (Shanghai, China). These siRNAs were transfected into NCI-H226/SK-MES-1 cells with Lipofectamine TM-2000 according to the manufacturer's protocol and diluted to 60 nM for the experiments.

### Plasmid and lentiviral expression vector construction

Human full-length TRIM8 or Wnt5a gene DNAs were cloned into pCMV2-GV146 plasmid, respectively. The pCMV2-GV146-TRIM8, pCMV2-GV146-Wnt5a or pCMV2-GV146-control plasmids were transiently transfected into NCI-H226/SK-MES-1 cells with Lipofectamine TM-2000, respectively. TRIM8 lentiviral expression vector was purchased from GenechemCompany Ltd (Shanghai, China). NCI-H226 cells were cultivated in a 12-well plate and transfected by using 1 mL viral stock for over 10 h, then, this transfected medium was replaced with normal RPMI-1640 medium.

### Luciferase reporter assay

The pmirGLO Dual-Luciferase expression plasmids were constructed by AuGCT DNA-SYN Biotechnology (Beijing, China). The wild type (WT) and mutated type (MT) binding sequences of miR-665 in the 3′-UTR of TRIM8 were cloned into the pmirGLO Dual-Luciferase expression plasmids, respectively. miR-665 mimics and reporter plasmids (WT or MT) were cotransfected into HEK293T cells, pmirGLO vector as their control. The HEK293T cells were collected 24 h after transfection. Next, the luciferase activity was detected by using the Dual-Luciferase Assay System (Promega, Madison, WI, USA).

### CCK8 cell proliferation assay

Human LUSC NCI-H226/SK-MES-1 cells were planted in 96-well plates at a density of 3000 cells/well and cultured for 24 h. Thereafter, the cells were transfected with miR-665 mimics, control, miR-665 inhibitor, negative control (NC), TRIM8 siRNA (60 nM), negative control siRNA (NC-siRNA, 60 nM), TRIM8 expression plasmid or control vector for 24, 48, 72 or 96 h, respectively. Each well was incubated with 10 μg Cell Counting Kit-8 solution (Dojindo, Shanghai, China) for 4 h. Cell proliferation was estimated by examining the absorbance at 450 nm with Varioskan Flash Spectral Scanning Multimode Reader (Waltham, MA, USA).

### Cell cycle analysis

The NCI-H226/SK-MES-1 cells at 1 × 10^5^ cells/well were seeded into 6-well plates in triplicate and transfected with miR-665 mimics, control, miR-665 inhibitor, negative control (NC), TRIM8 siRNA, NC-siRNA, TRIM8 expression plasmid or control vector for 24 h, respectively. These transfected cells were collected by trypsinization and fixed in 70% ice-cold ethanol at 4 °C. Next, 50 μg/mL propidium iodide (PI) and 10 U/mL RNaseA were added in the fixed cells and incubated 20 min for dyeing. The percentages of cell cycle (G1/G0, S and G2/M phases) were detected by using FACSAria flow cytometer (BD Biosciences, USA). Cell cycle was analyzed by using FACSort Cellquect software (BD Biosciences, USA).

### Apoptosis analysis

The NCI-H226/SK-MES-1 cells were cultivated in 6-well plates in triplicate and transfected with miR-665 mimics, control, miR-665 inhibitor, negative control (NC), TRIM8 siRNA, NC-siRNA, TRIM8 expression plasmid or control vector for 48 h, respectively. Cells (1 × 10^5^) were harvested and resuspended in 500 μL binding buffer. 5 μL Annexin-V-FITC and 5 μL PI (Invitrogen, Thermo Fisher Scientific, USA) were added in the cell suspension solution in dark condition 15 min for staining. Finally, early and late apoptosis were detected by using FACSAria flow cytometer (BD Biosciences, USA). Sub-G1 fractions also were measured with FACSAria flow cytometer.

### In vivo tumor xenograft model

To measure tumorigenicity, TRIM8 lentiviral expression vector transfected NCI-H226 cells were planted in 6-week-old male BALB/C nude mice. In brief, overexpressing TRIM8 cells or vector control cells (1 × 10^6^) were suspended with saline and injected subcutaneously into both posterior flanks of the mice (control group: n = 3, TRIM8 overexpression group: n = 3). Tumor sizes were measured with vernier calipers every 3 days for a total of 28 days. The length (L) and width (W) of tumour were used to figure the volume (V). V = (L × W^2^)/2. Lastly, the tumours were removed and weighed. The cancer tissues were promptly preserved at − 80 °C for the next experiment.

### TCF reporter assay

pTOPFLASH (including TCF sites and pRL-TK Renilla luciferase vector) were cotransfected with control vector, TRIM8 vector, NC-siRNA, or TRIM8 siRNA into NCI-H226/SK-MES-1 cells in a 96-well plate for 48 h. These cells were lysed by using Dual Luciferase kit (Promega, USA). The lysates were detected by using a 1420-Multilabel counter luminometer.

### RNA extraction and quantitative real-time PCR (qRT-PCR)

TRIzol reagent (Invitrogen, Carlsbad, CA, USA) was used to extract the RNA from LUSC clinical specimens and cell lines. miR-665 expression and TRIM8 mRNA expression were examined through using PrimeScript RT Reagent Kits and SYBR Premix Ex Taq II Kit (Takara Biotechnology, Takara, Dalian, China). The correlative primers of miR-665 and TRIM8 were purchased from Takara Biotechnology (Takara, Dalian, China). All reactions were performed in triplicate by using the iCycler iQ Multicolor Real-Time PCR System (Bio-Rad, CA, USA). The glyceraldehyde-3-phosphate dehydrogenase (GAPDH) was regarded as a control for TRIM8 mRNA. RNU6B (U6) was as a control for miR-665. The primers were as follows: miR-665 reverse-transcription primer (5′-GTCGTATCCAGTGCGTGTCGTGGAGTCGGCAATTGCACTGGATACGAC GGCCCCT-3′), miR-665 (F: 5′-ATCCAGTGCGTGTCGTG-3′; R: 5′-TGCT ACCAGGAGGCTGA-3′); U6 reverse-transcription primer (5′-CGCTTCACGAATTTGCGTGTCAT-3′), U6 (F: 5′-GCTTCGGCAGCACATATACTAAAAT-3′; R: 5′-CGCTTCACGAATTTGCGTGTCAT-3′); TRIM8 (F: 5′- CGTGGAGATCCGAAGGAATGA-3′; R: 5′-CAGGCGCTTGTCTGACTCG-3′), GAPDH (F: 5′-GCCACATCGCTCAGACAC-3′; R: 5′-GCCCAATACGACCAAATCC-3′). miR-665 reactions conditions: 95 °C for 15 min, followed by 40 cycles at 95 °C for 5 s, 58 °C for 30 s, and 72 °C for 30 s. TRIM8 reactions conditions: 95 °C for 10 min, followed by 40 cycles at 95 °C for 10 s, 60 °C for 20 s, and 72 °C for 30 s. The 2^−ΔΔCt^ method was applied in the qRT-PCR analysis.

### Western blot analysis

LUSC clinical specimens, cultured cells and grafting tumor tissues were lysed for extracts through using RIPA lysis buffer (Invitrogen, Carlsbad, CA, USA) supplemented with protease inhibitors (Roche, Indianapolis, IN, USA). Equal amounts of protein lysates was separated with 10% SDS polyacrylamide gels and transferred to polyvinylidene fluoride (PVDF) membranes. The membranes were interdicted by using 5% nonfat milk in Tris-buffered saline (TBST, including 10 mM Tris–HCl and 0.05% Tween 20). Next, the primary antibodies were used to incubate the membranes overnight at 4 °C. The corresponding secondary antibodies were used to incubate the membranes for 1 h. The primary antibodies were as follow: mouse monoclonal anti-TRIM8 (1:2000; Abcam Ltd., UK), rabbit monoclonal anti-Wnt5a (1:2000, Cell Signaling, MA, USA), rabbit polyclonal anti-β-Catenin (1:1000, Cell Signaling, MA, USA), mouse monoclonal anti-c-Myc (1:1000; Cell Signaling, MA, USA), mouse monoclonal anti-Caspase-3 (1:1000; Cell Signaling, MA, USA), and mouse monoclonal anti-Cyclin D1 (1:2000, Santa Cruz, CA, USA), mouse monoclonal anti-PCNA (1:1000, Santa Cruz, CA, USA), mouse monoclonal anti-Lamin A (1:2000, Santa Cruz, CA, USA), mouse monoclonal anti-GAPDH (1:3000, Santa Cruz, CA, USA). The ECL reagent (Amersham, USA) was used to incubate the membranes. Then, the intensity of luminescent signals was recorded and analyzed through using Syngene GBox (Syngene, Cambridge, UK).

### Statistical analysis

Statistical analysis was fulfilled with SPSS 25.0 software (SPSS, Inc., Chicago, IL, USA). All experiments were fulfilled at least in triplicates. The quantitative data were presented as mean ± SEM. Significant between-group differences were estimated with one-way ANOVA or Wilcoxon test, as appropriate. The logistic regression analysis was used to confirm the cut-off value of miR-665. Spearman’s correlation analysis was used to assess the relationship between miR-665 and TRIM8 in human LUSC tissues. The χ^2^ test was used to analyze the relationship between miR-665 expression and clinicopathological parameters. p < 0.05 was considered statistically significant.

## Results

### miR-665 is frequently upregulated in LUSC tissues and is correlated with clinicopathological parameters

**Fig. 1:**
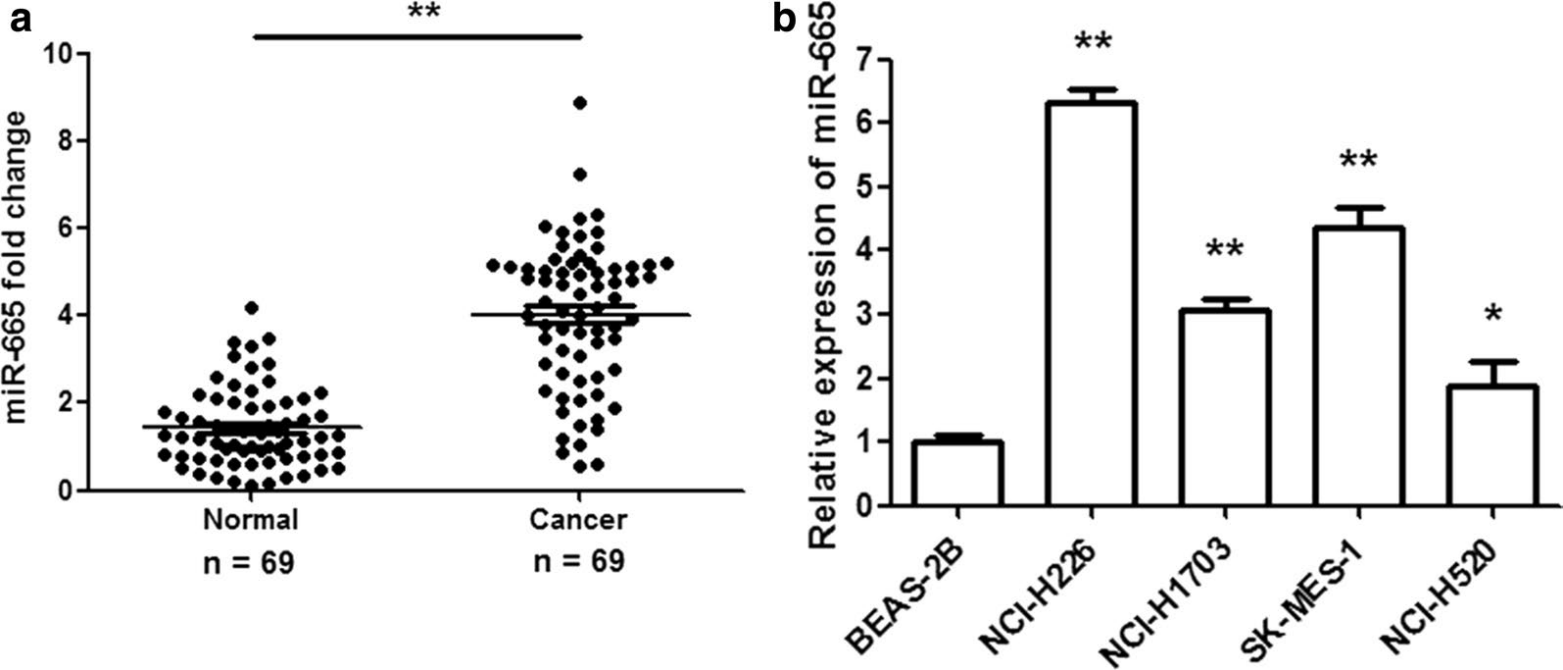
miR-665 levels are upregulated in LUSC tissues and cells. **a** The miR-665 expression levels were remarkably higher in LUSC tissues than in adjacent normal tissues. **b** The miR-665 expressions were significantly increased in LUSC cell lines (NCI-H226, NCI-H1703, SK-MES-1 and NCI-H520) compared to normal human lung epithelial cells (BEAS-2B). n = 3, *p < 0.01, **p < 0.001

To explore the function of miR-665 in LUSC, the expression of miR-665 was measured in clinical tissues (69 paired LUSC sample tissues and adjacent normal tissues) and LUSC cell lines by using qRT-PCR assay. The results revealed that the miR-665 levels were remarkably higher in LUSC tissues than in normal tissues (Fig. 1a; p < 0.001). Further investigation showed that miR-665 expressions were significantly upregulated in 81.2% (56/69) of the LUSC tissues compared to the adjacent normal tissues. Then, we analyzed the relationship between miR-665 expression levels and the clinicopathological parameters of LUSC patients. High miR-665 expression was associated with differentiation (Well: 65.4%; moderate-poor: 90.7%), tumor size (≥ 3 cm: 94.0%; < 3 cm: 47.4%) and TNM stage (I + II: 71.9%; III + IV: 89.2%) (Table 1). Nevertheless, miR-665 expression was not correlated with sex, age, smoke and metastasis. Moreover, the expression of miR-665 was observably increased in human LUSC cell lines (NCI-H226, NCI-H1703, SK-MES-1 and NCI-H520) compared to normal human lung epithelial cells (BEAS-2B) (Fig. 1b; p < 0.01). These findings suggested that miR-665 might play as an oncogene in human LUSC and be a potential effective biomarker.

**Table 1:** Relationship between miR-665 expression and clinicopathological parameters in LUSC patients (n = 69)

Parameter	All patients	miR-665 expression		*p* value
		High (n = 56)	Low (n = 13)	
Sex				0.726
Male	57	46	11	
Female	12	10	2	
Age (years)				0.805
≥ 60	25	20	5	
< 60	44	36	8	
Smoke				0.561
No	42	35	7	
Yes	27	21	6	
Differentiation				0.013*
Moderate–poor	43	39	4	
Well	26	17	9	
Metastasis				0.237
Yes	49	41	8	
No	20	15	5	
Tumor size (cm)				0.001*
≥ 3	50	47	3	
< 3	19	9	10	
TNM stage				0.039*
I + II	32	23	9	
III + IV	37	33	4	

The cut-off value of miR-665 was 1.52. *p < 0.01

### miR-665 promotes LUSC cell proliferation and represses apoptosis

**Fig. 2:**
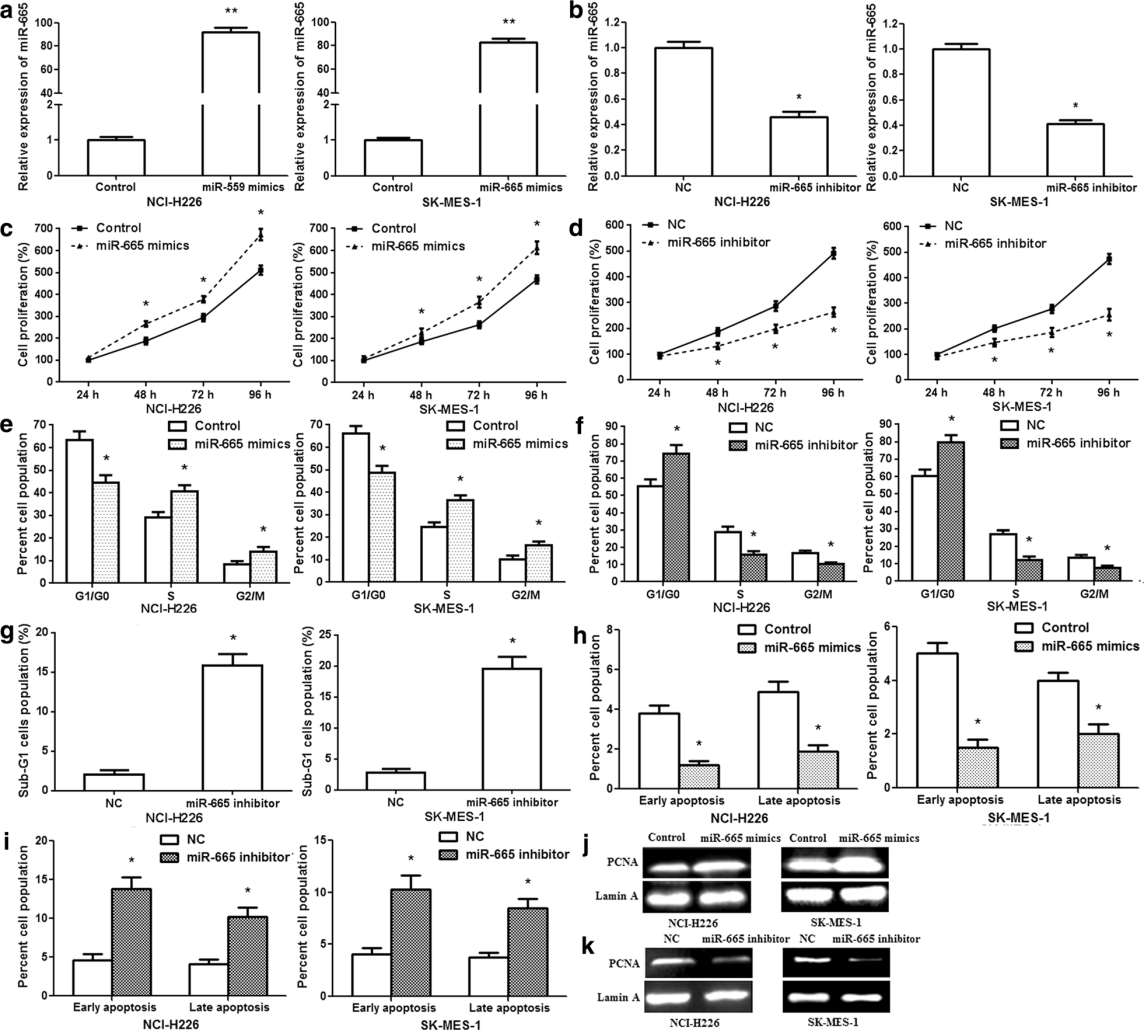
miR-665 enhances LUSC NCI-H226/SK-MES-1 cell growth and reduces apoptosis. **a** miR-665 expressions increased in NCI-H226/SK-MES-1 cells after transfection with the miR-665 mimics. **b** miR-665 expressions decreased after transfection with the miR-665 inhibitor. **c** CCK8 results showed that miR-665 mimics facilitated LUSC cell proliferation at 48, 72 and 96 h after transfection. **d** CCK8 results showed that miR-665 inhibitor repressed LUSC cell proliferation at 48, 72 and 96 h after transfection. **e** Flow cytometry analysis of cell cycle. The proportion of cells changed in the G0/G1, S and G2/M stages after transfection with miR-665 mimics. **f** The ratio of cells changed in the G0/G1, S and G2/M stages after transfection with miR-665 inhibitor. **g** The percentage of sub-G1 cells population changed after transfection with miR-665 inhibitor. **h** Flow cytometry analysis of cell apoptosis. The ratios of early and late apoptosis reduced after transfection with miR-665 mimics. **i** The ratios of early and late apoptosis increased after transfection with miR-665 inhibitor. **j** PCNA protein expression upregulated after transfection with the miR-665 mimics. **k** PCNA protein expression downregulated after transfection with the miR-665 inhibitor. n = 3, *p < 0.01, **p < 0.001.

NCI-H226/SK-MES-1 cells were transfected with miR-665 mimics, control, miR-665 inhibitor, or negative control. qRT-PCR was fulfilled to examine the miR-665 expression of after transfection. The results revealed that miR-665 expression was observably upregulated in cells transfected with the miR-665 mimics compared to that in cells transfected with control; while miR-665 expression was significantly downregulated in cells transfected with miR-665 inhibitor compared to that in cells transfected with negative control (Fig. 2a, b; p < 0.01). The CCK8 assay revealed that miR-665 mimics remarkably facilitated the proliferation of NCI-H226/SK-MES-1 cells after transfection (Fig. 2c; p < 0.01), whereas miR-665 inhibitor significantly restrained NCI-H226/SK-MES-1 cell growth after transfection (Fig. 2d; p < 0.01). Moreover, miR-665 mimics promoted the proliferating cell nuclear antigen (PCNA) expression after transfection and miR-665 inhibitor suppressed the PCNA expression (Fig. 2j, k). Cell cycle assay showed that miR-665 mimics remarkably reduced the proportion of G1/G0 phase and enhanced the proportion of S and G2/M phase in NCI-H226/SK-MES-1 cells (Fig. 2e; p < 0.01); nevertheless, miR-665 inhibitor led to the marked accumulation in the G1/G0 phase and the decreasing of proportion of S and G2/M phase (Fig. 2f, Additional file 1: Fig. S1a, b; p < 0.01). miR-665 inhibitor increased the percentage of sub-G1 cells population (Fig. 2g; p < 0.01). Apoptosis assay showed that miR-665 mimics significantly decreased the percentage of early and late apoptosis (Fig. 2h; p < 0.01); however, miR-665 inhibitor observably increased the percentage (Fig. 2i, Additional file 1: Fig. S1c, d; p < 0.01). The results demonstrated that miR-665 facilitated LUSC cell proliferation and cell cycle G1-S transition, and restrained apoptosis.

### TRIM8 is a direct target gene of miR-665

**Fig. 3:**
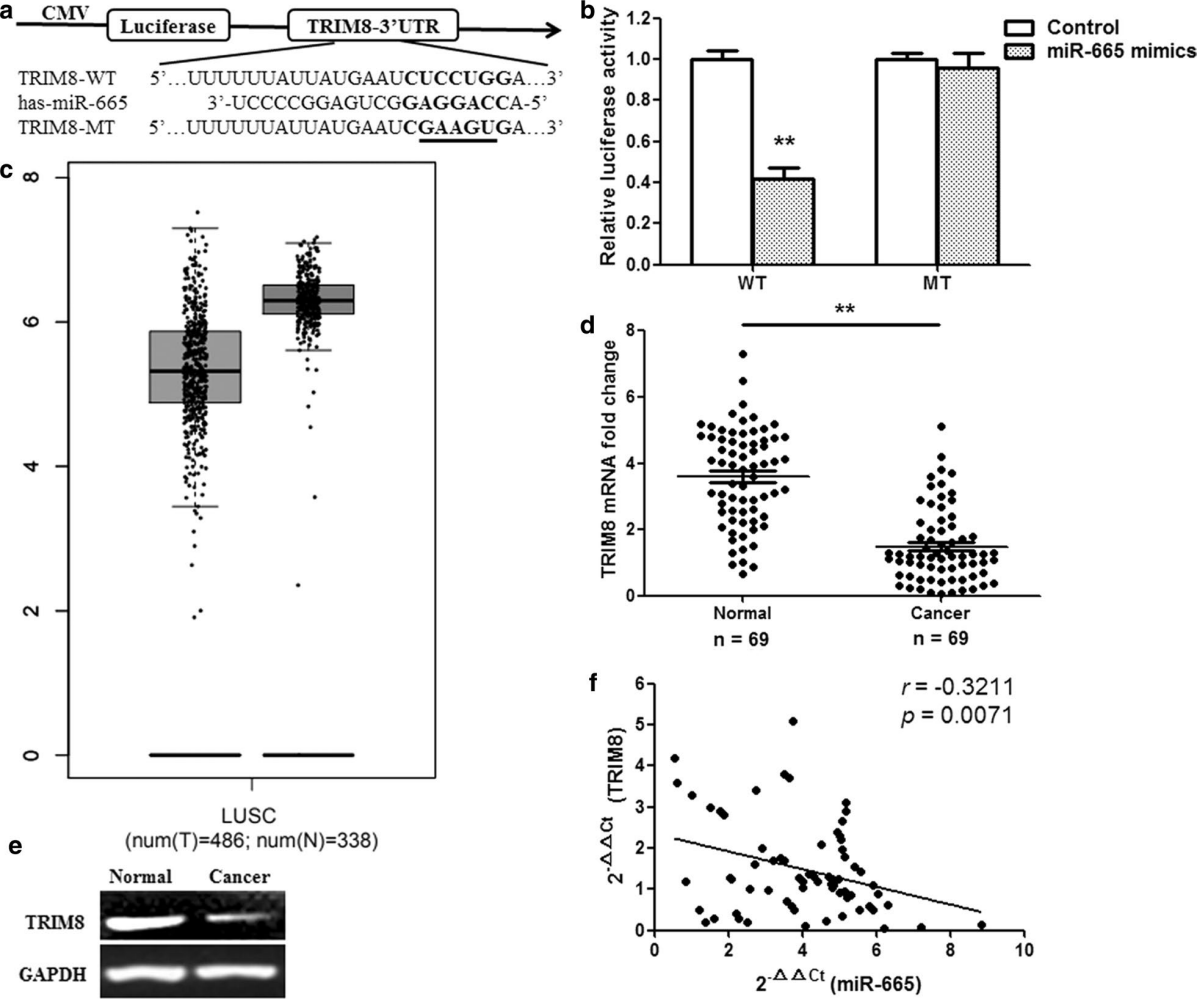
miR-665 downregulates TRIM8 expression through targeting its 3′-UTR. **a** Bioinformatics predicted the binding sites of miR-665 in the 3′-UTR of TRIM8. **b** Luciferase activity was detected by using the dual-luciferase assay. **c** TCGA data showed the TRIM8 expressions in human LUSC tissues. **d** qRT-PCR assay revealed the TRIM8 mRNA expressions in LUSC tissues. **e** TRIM8 protein levels were measured by Western blotting. **f** The expressions of miR-665 and TRIM8 were negatively correlated. The 2^−ΔΔCt^ values of miR-665 and TRIM8 mRNA were subjected to a Spearman correlation analysis (r = − 0.3211, n = 69, p = 0.0071). n = 3, *p < 0.01, **p < 0.001

TRIM8 was selected as the target gene of miR-665 from a large number of possible target genes by using bioinformatic databases (miRBase and TargetScanHuman). There was a binding site for miR-665 in the 3′-UTR of the TRIM8 mRNA ranging from 172 to 195 bp (Fig. 3a). To confirm the target relationship between miR-665 and TRIM8, a dual-luciferase reporter system including the wild-type (WT) and mutant-type (MT) 3′-UTR of TRIM8 was fulfilled. HEK293T cells were cotransfected with WT 3′-UTR or MT 3′-UTR constructs and miR-665 mimics or control. The results showed that miR-665 mimics induced a significant reduction in luciferase activity of the WT 3′-UTR (TRIM8) binding site (p < 0.01), while miR-665 mimics failed to suppress the luciferase activity of the reporter construct containing the MT 3′-UTR (TRIM8) binding site, indicating that miR-665 directly targets the 3′-UTR of TRIM8 (Fig. 3b). The Cancer Genome Atlas (TCGA) data showed that TRIM8 expression significantly downregulated in LUSC tissues than in normal tissues (Fig. 3c; p < 0.01). Then, we detected TRIM8 expressions at the mRNA and protein levels. The results revealed that TRIM8 expressions were remarkably decreased at both the mRNA and protein levels in LUSC tissues compared to in normal tissues (Fig. 3d, e; p < 0.01). A significant inverse correlation was identified between miR-665 expression and TRIM8 mRNA level in the LUSC specimens (Fig. 3f; n = 69, r = − 0.3211, p < 0.01, Spearman correlation analysis). The data demonstrated that miR-665 post-transcriptionally regulated TRIM8 by directly targeting its 3′-UTR in LUSC cells.

### miR-665 regulates LUSC cell growth and apoptosis via regulation of the Wnt5a/β-Catenin and Caspase-3 signaling pathways by targeting TRIM8

**Fig. 4:**
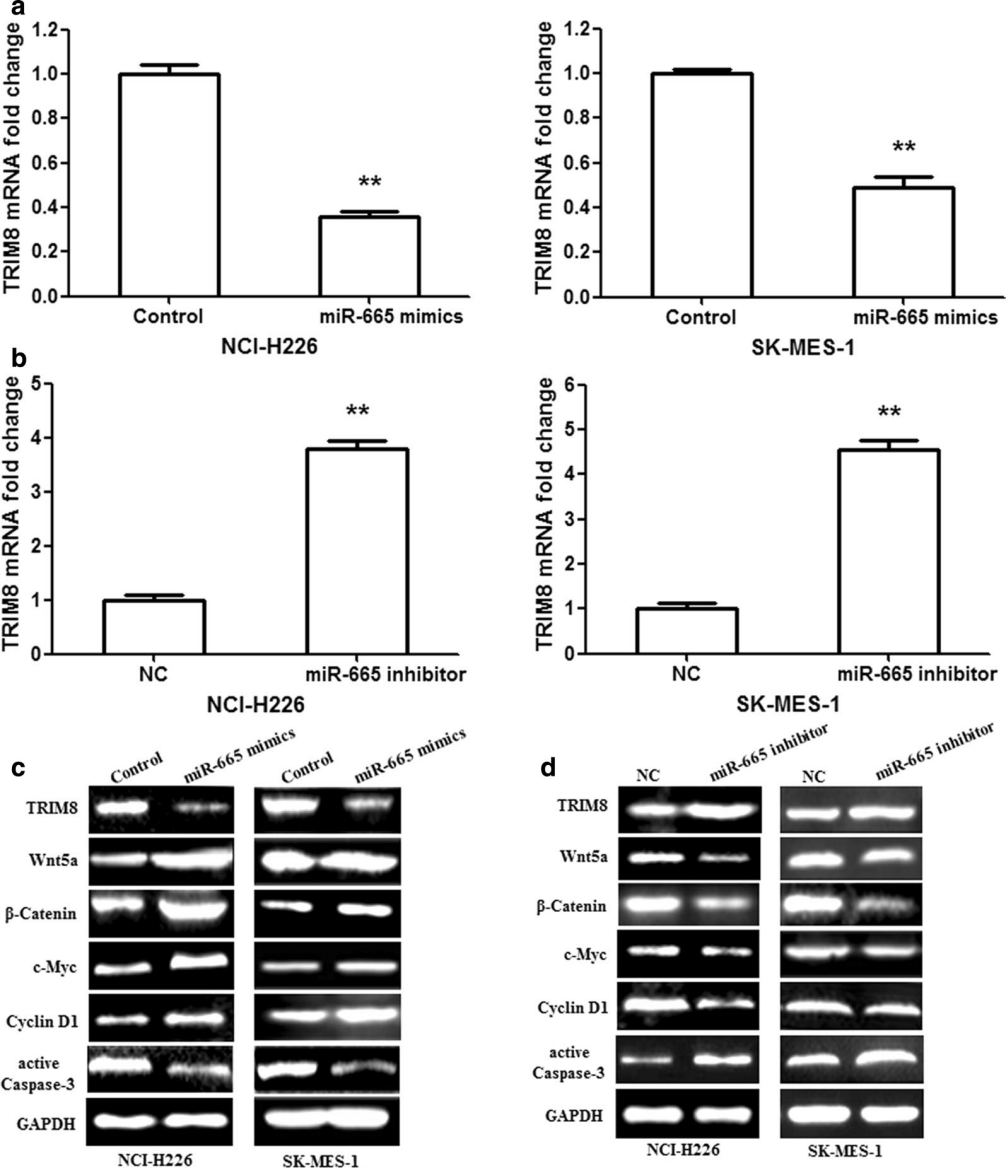
miR-665 regulates the Wnt5a/β-Catenin and Caspase-3 signaling pathways in human LUSC cells by targeting TRIM8. **a** TRIM8 mRNA was measured in NCI-H226/SK-MES-1 cells after transfection with the miR-665 mimics. **b** TRIM8 mRNA was determined in NCI-H226/SK-MES-1 cells after transfection with the miR-665 inhibitor. **c** miR-665 mimics facilitated the expression of Wnt5a, β-Catenin, c-Myc and Cyclin D1 proteins, and repressed active Caspase-3 protein expression. **d** miR-665 inhibitor suppressed the expression of Wnt5a, β-Catenin, c-Myc and Cyclin D1 proteins, and enhanced active Caspase-3 protein expression. n = 3, **p < 0.001

TRIM8 mRNA and protein expressions were measured after transfection with miR-665 mimics or inhibitor. miR-665 mimics observably downregulated the mRNA expression of TRIM8 in NCI-H226/SK-MES-1 cells, while miR-665 inhibitor remarkably upregulated TRIM8 mRNA expression (Fig. 4a, b; p < 0.01). The similar results were also observed in protein expressions (Fig. 4c, d). To further explore the underlying mechanisms of miR-665-regulated LUSC cell proliferation and apoptosis, we detected the correlation factor expression in the Wnt5a/β-Catenin signaling pathway. The results showed that miR-665 mimics increased Wnt5a, β-Catenin, c-Myc and Cyclin D1 protein expressions, and decreased active Caspase-3 protein expression in NCI-H226/SK-MES-1 cells (Fig. 4c). Nevertheless, miR-665 inhibitor restrained Wnt5a, β-Catenin, c-Myc and Cyclin D1 protein expressions, and enhanced active Caspase-3 protein expression (Fig. 4d). These findings suggested that miR-665 might affect LUSC cell proliferation and apoptosis through regulating Wnt5a/β-Catenin and Caspase-3 signaling pathways.

### TRIM8 silencing generates similar effects as of miR-665 mimics in NCI-H226/SK-MES-1 cells

**Fig. 5:**
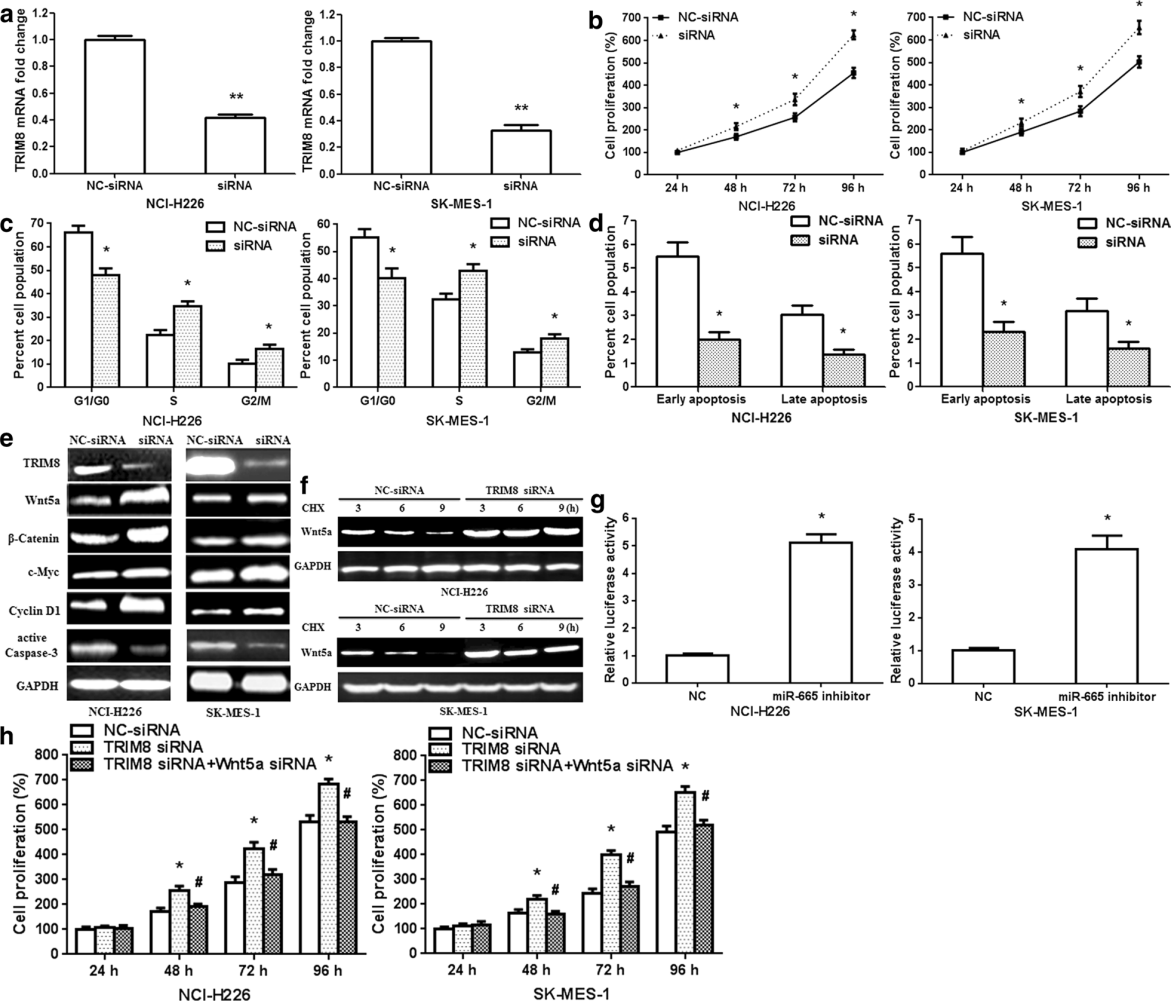
TRIM8 siRNA facilitates human LUSC cell proliferation and inhibits apoptosis. **a** qRT-PCR showed the silencing efficiency of TRIM8 siRNA in NCI-H226/SK-MES-1 cells at 24 h after transfection. **b** CCK8 assay revealed that TRIM8 siRNA promoted the proliferation of NCI-H226/SK-MES-1 cells. **c** Flow cytometric analysis showed that G0/G1 phase cells reduced after TRIM8 siRNA transfection, while S and G2/M phase cells were increased. **d** Apoptosis assay revealed the proportion of early and late apoptosis after TRIM8 siRNA transfection. **e** Wnt5a, β-Catenin, c-Myc, Cyclin D1 and active Caspase-3 protein expressions were detected at 48 h after TRIM8 siRNA transfection. **f** TRIM8 siRNA inhibited Wnt5a degradation in NCI-H226/SK-MES-1 cells. 10 μg/mL cycloheximide (CHX) was used to suppress de novo Wnt5a biosynthesis. **g** TRIM8 siRNA affected TOPFLASH TCF-reporter construct activity in NCI-H226/SK-MES-1 cells. **h** CCK8 assay revealed cell proliferation after co-transfection with TRIM8 siRNA and Wnt5a siRNA in NCI-H226/SK-MES-1 cells. n = 3, *p < 0.01, **p < 0.001, as compared with NC-siRNA group; ^#^p < 0.01, as compared with TRIM8 siRNA group

Since miR-665 mimics modulated human LUSC cell proliferation, cell cycle and apoptosis, TRIM8 was affirmed as a direct target of miR-665, thus, TRIM8 was silenced in NCI-H226/SK-MES-1 cells by siRNA to confirm its involvement in the pro-tumor roles of miR-665. qRT-PCR showed that TRIM8 mRNA levels were specifically knocked down in NCI-H226/SK-MES-1 cells after transfection with TRIM8 siRNA (Fig. 5a; p < 0.001). TRIM8 knockdown markedly enhanced NCI-H226/SK-MES-1 cell proliferation after transfection with TRIM8 siRNA (Fig. 5b; p < 0.01). Silencing TRIM8 significantly reduced the G1/G0 phase population and enhanced the S and G2/M phase populations in LUSC cells (Fig. 5c, Additional file 1: Fig. S2a; p < 0.01). Moreover, silencing TRIM8 also observably restrained LUSC cell apoptosis (Fig. 5d, Additional file 1: Fig. S2b; p < 0.01). Then, we analyzed silencing efficiency of TRIM8 siRNA in protein level. The results revealed that TRIM8 protein expression was downregulated in LUSC cells by siRNA. In addition, TRIM8 siRNA upregulated Wnt5a, β-Catenin, c-Myc and Cyclin D1 protein expressions, and downregulated active Caspase-3 protein expression in NCI-H226/SK-MES-1 cells (Fig. 5e). To verify that TRIM8 affects Wnt5a stability, we treated GC cells with cycloheximide after transfection with TRIM8 siRNA. The results showed that TRIM8 siRNA inhibited Wnt5a degradation in NCI-H226/SK-MES-1 cells (Fig. 5f). The luciferase activity of TOPFLASH TCF-reporter remarkably increased in TRIM8 siRNA group compared with NC-siRNA group (Fig. 5g). This finding confirmed that TRIM8 regulated Wnt5a/β-Catenin signaling pathway. CCK8 assay demonstrated that Wnt5a siRNA attenuated the effect of TRIM8 siRNA on GC cell proliferation (Fig. 5h).

### TRIM8 overexpression suppresses LUSC cell proliferation in vitro and in vivo

**Fig. 6:**
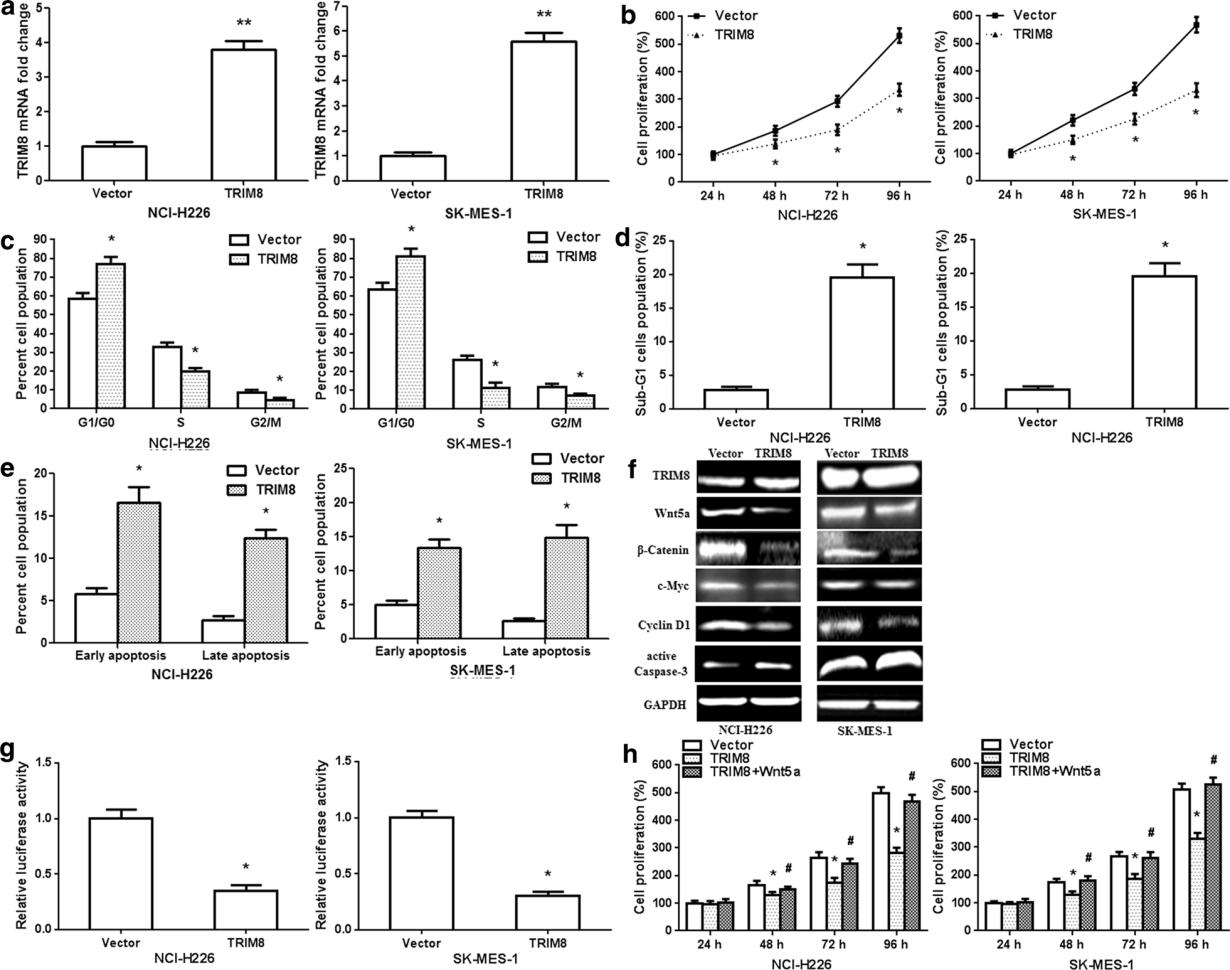
TRIM8 overexpression suppresses the proliferation of human LUSC cells. **a** TRIM8 overexpression increases TRIM8 mRNA levels in NCI-H226/SK-MES-1 cells. **b** CCK8 assay showed that TRIM8 overexpression repressed NCI-H226/SK-MES-1 cell proliferation. **c** Cell cycle was detected in NCI-H226/SK-MES-1 cells at 24 h after transfection. **d** The percentage of sub-G1 cells population changed after transfection with TRIM8 overexpression vector. **e** Apoptosis was measured in NCI-H226/SK-MES-1 cells at 48 h after transfection. **f** Wnt5a, β-Catenin, c-Myc, Cyclin D1 and active Caspase-3 protein levels were detected after TRIM8 overexpression vector transfection. **g** TRIM8 overexpression affected TOPFLASH TCF-reporter construct activity in NCI-H226/SK-MES-1 cells. **h** CCK8 assay revealed cell proliferation after co-transfection with TRIM8 and Wnt5a overexpression vectors in NCI-H226/SK-MES-1 cells. n = 3, *p < 0.01, **p < 0.001, as compared with Empty Vector-transfected cells; ^#^p < 0.01, as compared with TRIM8 overexpression Vector-transfected cells

To further identify that miR-665 played an oncogenic role via TRIM8, we constructed TRIM8 overexpression vector. In NCI-H226/SK-MES-1 cells, TRIM8 overexpression vector significantly increased the TRIM8 mRNA expression (Fig. 6a; p < 0.001). CCK8 assay showed that TRIM8 overexpression observably suppressed NCI-H226/SK-MES-1 cell proliferation (Fig. 6b; p < 0.01). TRIM8 overexpression remarkably increased the ratio of G1/G0 phase and decreased the ratio of S and G2/M phase (Fig. 6c, Additional file 1: Fig. S3a; p < 0.01). TRIM8 overexpression increased the percentage of sub-G1 cells population (Fig. 6d; p < 0.01). In addition, TRIM8 overexpression also significantly facilitated early and late apoptosis (Fig. 6e, Additional file 1: Fig. S3b; p < 0.01). Then, TRIM8 protein expression efficiency was further detected. TRIM8 protein expression upregulated in LUSC cells after transfection with TRIM8 expression vector. TRIM8 overexpression decreased Wnt5a, β-Catenin, c-Myc and Cyclin D1 protein expressions, and increased active Caspase-3 protein expression in LUSC cells (Fig. 6f). The luciferase activity of TOPFLASH TCF-reporter remarkably decreased in TRIM8 overexpression vector group compared with empty vector group (Fig. 6g). The result confirmed that TRIM8 regulated Wnt5a/β-Catenin signaling pathway. CCK8 assay showed that Wnt5a overexpression reversed the effect of TRIM8 overexpression on GC cell proliferation (Fig. 6h).

**Fig. 7:**
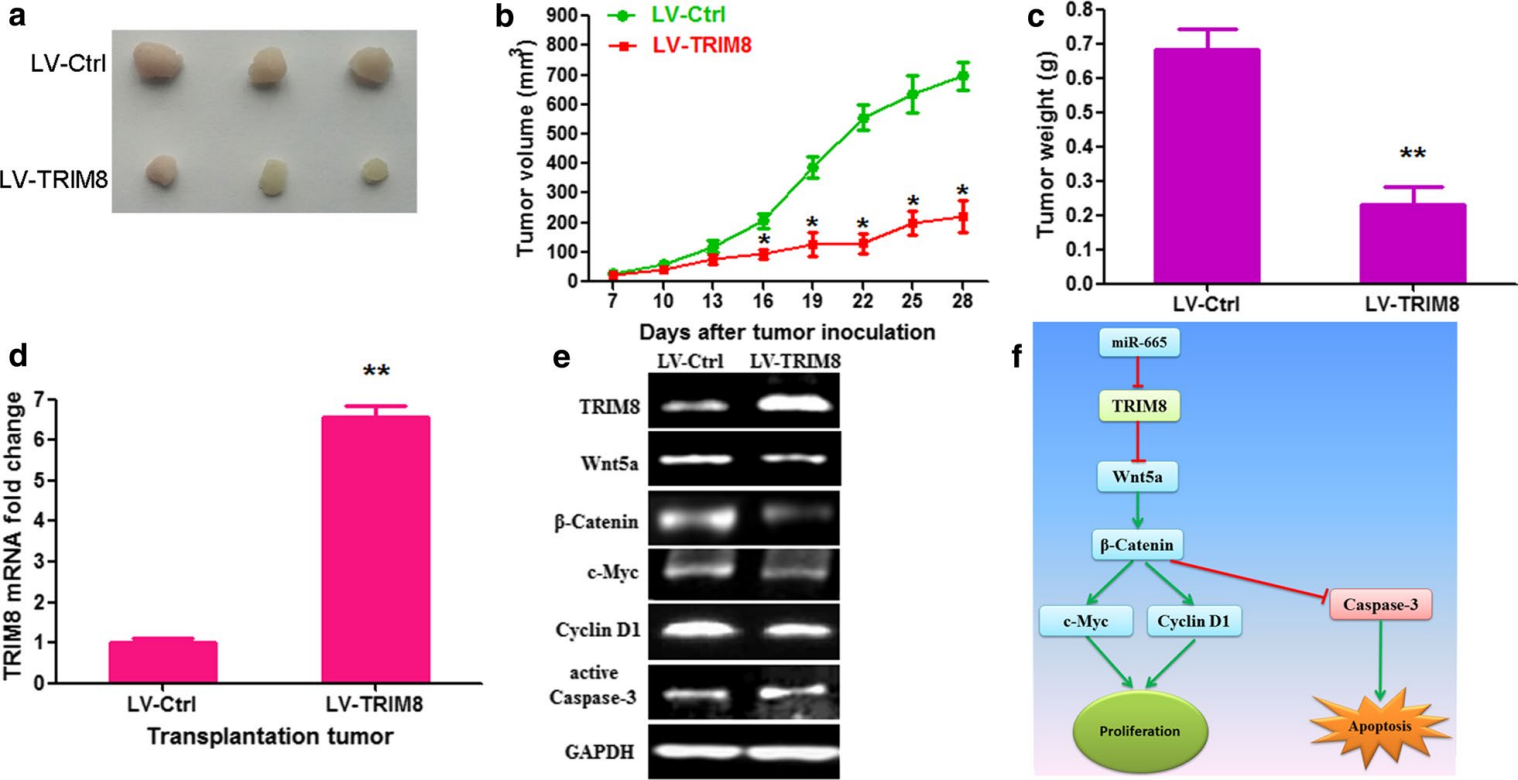
TRIM8 overexpression restrains LUSC tumor growth in vivo. **a** Morphology of excised tumors from nude mice were observed 28 days after being injected with LV-TRIM8-infected or LV-Ctrl-infected NCI-H226 cells. **b** Growth curves of tumor volume were generated every 3 days for 21 days. **c** Tumor weights were measured at day 28 after the injection. **d** TRIM8 mRNA expression in transplantation tumor was examined by qRT-PCR. **e** TRIM8 modulated the protein expressions of Wnt5a, β-Catenin, c-Myc, Cyclin D1 and active Caspase-3 in vivo. **f** Proposed model for the effects of TRIM8 on LUSC progression. miR-665 promotes human LUSC cell proliferation and suppresses apoptosis through regulation of the Wnt5a/β-Catenin and Caspase-3 signaling pathways by targeting TRIM8. n = 3, *p < 0.01, **p < 0.001

In an attempt to further analyze the function of TRIM8 in LUSC progression in vivo, we constructed TRIM8 overexpression lentiviral vector and produced a stable NCI-H226 cell clone. LV-TRIM8-infected and LV-Ctrl-infected NCI-H226 cells were planted subcutaneously into both posterior flanks of the nude mouse and monitored tumor growth 4 weeks. The results of weights and sizes of tumors showed that tumor growth was remarkably restrained by LV-TRIM8 compared to LV-Ctrl (Fig. 7a–c; p < 0.01). The TRIM8 mRNA expression in LV-TRIM8 cancers was about 6.6-fold higher than that in LV-Ctrl cancers (Fig. 7d; p < 0.01). The TRIM8 protein expression also increased in LV-TRIM8 cancers (Fig. 7e). TRIM8 overexpression downregulated the protein expressions of Wnt5a, β-Catenin, c-Myc and Cyclin D1, and upregulated the protein expression of active Caspase-3 in LV-TRIM8 cancers (Fig. 7e). Based on these findings, we concluded that miR-665 could modulate human LUSC cell proliferation and apoptosis through regulation of the Wnt5a/β-Catenin and Caspase-3 signaling pathways by targeting TRIM8.

## Discussion

In the last decade, numerous studies have demonstrated that miRNAs play as oncogenes or tumor suppressor genes through modulating target gene in all kinds of tumors. Because a single miRNA can regulate multiple gene mRNAs, the disorder of miRNAs can result in the imbalance of RNA network in oncogenesis and development [24]. A plethora of studies have found that miRNAs are involved in multiple steps of tumor diseases, including growth, migration, invasion, metabolism and metastasis [25, 26]. Currently, only a few studies have found that miR-665 play an important role in some cancers [18, 21]. A previous study reveals that miR-665 expression is correlated with breast cancer survival and miR-665 expression predicts poor and promotes tumor metastasis by targeting NR4A3 [19]. It is reported that miR-665 promotes cell proliferation and suppresses apoptosis in acute lymphoblastic leukemia by suppressing EXT1 [20]. Zhou et al. have also found that miR-665 facilitates the proliferation of ovarian cancer cells via activating MAPK/ERK signaling by targeting SRCIN1 [21]. However, the functions and mechanism of miR-665 in LUSC are still not understood. In this study, our results showed that miR-665 expressions were remarkably upregulated in both human LUSC specimen tissues and cell lines, and correlated with clinicopathological parameters. miR-665 mimics enhanced LUSC cell proliferation and cell cycle G1-S phase transition, and repressed cell apoptosis in vitro. miR-665 inhibitor restrained LUSC cell growth and promoted cell apoptosis. In a word, miR-665 plays as an oncogenic function in human LUSC and might be a new diagnostic marker and a potential new target for LUSC therapy.

The further research confirmed that TRIM8 is a direct target gene of miR-665. The TRIM protein family was consisted of multidomain ubiquitin E3 ligases, including a RING finger, one or two B-box and a Coiled Coil region [27, 28]. Previous studies show that TRIM proteins act as key roles in multiple biological processes, such as cell proliferation, cycle, apoptosis, metabolism, carcinogenesis and immune response [29, 30]. In different cellular context, TRIM proteins may exhibit a dual role either as cancer suppressor gene or oncogene [31, 32]. For example, TRIM8 shows multiple functions in embryonic development and tumours [33, 34]. Recently, it is reported that TRIM8 inhibits gliomagenesis through the regulation of JAK–STAT signaling pathway [35]. TRIM8 overexpression blocks cell proliferation of clear cell Renal Cell Carcinoma [23]. TRIM8 expression was downregulated in anaplastic thyroid cancer tissues and suppresses cell proliferation [36]. However, the function of TRIM8 remains unclear in LUSC. Our results showed that TRIM8 expression significantly decreased in LUSC tissues. A significant negative correlation was identified between miR-665 expression and TRIM8 mRNA in the LUSC specimens. The dual-luciferase reporter assay demonstrated that TRIM8 was a direct target gene of miR-665. miR-665 mimics repressed TRIM8 expression at both mRNA and protein levels in human LUSC cells. Silencing of TRIM8 enhanced LUSC cell proliferation and cell cycle G1-S phase transition, and suppressed apoptosis. On the contrary, overexpression of TRIM8 restrained cell proliferation in vitro and in vivo, and promoted apoptosis. Gene expression modulation is a molecular network regulation system and involves in multiple regulatory factors. TRIM8 expression is also regulated in GC by other miRNAs or regulators. Therefore, TRIM8 expression downregulation could be partly mediated by miR-665. The findings suggest that miR-665 plays as an oncogene in LUSC by targeting TRIM8.

The Wnt signaling pathway is a canonical signal transduction system in physiological and pathological processes. It is intimately correlated to a number of crucial biological functions, such as stress, embryonic development, injury, inflammation, immunity, metabolism, growth, repair, and apoptosis [37]. Wnt5a belongs to the Wnt protein family and is involved in both classical and nonclassical Wnt signaling pathways [38, 39]. Previous studies have shown that Wnt5a/β-catenin signaling participates in the cell survival, proliferation, cycle, migration, invasion, metastasis, apoptosis, and multidrug resistance of various cancers [40]. Wnt5a/β-catenin signaling pathway regulates the important downstream genes including BIRC5, CyclinD1, c-Myc, and Axin2 [41]. TRIM8 owns an E3 ubiquitin ligase activity conferred via its RING domain [42]. TRIM8 participates in various pathological processes by interacting with and modulating E3-mediated ubiquitination plentiful substrates, such as PIAS3, p53, and SOCS1 [43–45]. The present study demonstrated that miR-665 mimics and silencing TRIM8 promoted LUSC cell proliferation and induced cell cycle G1-S phase transition through facilitating the Wnt5a/β-catenin signaling pathway, whereas miR-665 inhibitor and TRIM8 overexpression suppressed cell proliferation and led to G1-S phase transition arrest via repressing the Wnt5a/β-catenin signaling pathway. Our findings suggest that miR-665 may promote LUSC cell proliferation and cycle G1-S phase transition via modulating the Wnt5a/β-catenin signaling pathway by targeting TRIM8 (Fig. 7f).

Caspases are a family of cysteine proteases acting as the critical functions in apoptosis and inflammation [46]. Apoptosis signaling cascades are referred to plenty of pathways in endoplasmic reticulum and mitochondria. Among them, Caspase-3 is a canonical apoptotic executioner and induces apoptosis by cleaving other functionally pivotal proteins within the cell [47]. Plenty of anticancer therapies can result in tumor cell death through activating Caspase-3, such as chemotherapy, radiotherapy, and immunotherapy. Some studies have found that TRIM8 can play a variety of biological functions by regulating the protein expressions of STAT3, Caspase-3, IRF3, IRF7, c-MYC, SOX2, NESTIN and CD133 [48–50]. Wnt/β-catenin pathway plays great roles in anti-apoptosis, hence, the variation of caspase-3 could be caused by the status of Wnt/β-catenin. The results showed that miR-665 mimics or silencing TRIM8 restrained LUSC cell apoptosis via inhibiting Caspase-3 signaling pathway; while miR-665 inhibitor and TRIM8 overexpression promote apoptosis through activating Caspase-3 signaling pathway. Our findings implied that miR-665 could respress LUSC cell apoptosis through regulating Caspase-3 signaling pathway by targeting TRIM8 (Fig. 7f).

## Conclusions

In short, the current study demonstrates that miR-665 acts as an oncogene in human LUSC. It is discovered that miR-665 is observably upregulated in human LUSC specimen tissues. miR-665 facilitates LUSC cell proliferation and cell cycle transition by regulation of the Wnt5a/β-Catenin signaling pathway and represses cell apoptosis via modulation of Caspase-3 signaling pathway by targeting TRIM8. These findings indicate that miR-665 might be a potential new target for LUSC therapy.

## Supplementary Information

**Additional file 1: Fig. S1.** The raw charts of flow cytometry. a Cell cycle was measured after transfection with the miR-665 mimics. b Cell cycle was detected after transfection with the miR-665 inhibitor. c Apoptosis was measured after transfection with the miR-665 mimics. d Apoptosis was examined after transfection with the miR-665 inhibitor. **Fig. S2.** The raw charts of flow cytometry. a Cell cycle was detected after TRIM8 siRNA transfection. b Apoptosis was measured after TRIM8 siRNA transfection. **Fig. S3.** The raw charts of flow cytometry. a Cell cycle was examined after TRIM8 overexpression vector transfection. b Apoptosis was measured after TRIM8 overexpression vector transfection.

## Data Availability

The datasets used in present study are available from the corresponding author upon reasonable request.

## Abbreviations

LUSC: Lung squamous cell carcinoma
miRNAs: MicroRNAs
miR-665: MicroRNA-665
NSCLC: Non-small cell lung cancer
MT: Mutation type
qRT-PCR: Quantitative real-time PCR
siRNAs: Small interfering RNAs
TRIM8: Tripartite motif 8
WT: Wild type
3′-UTR: 3′-Untranslated region
