# Supplementary figures and images for: MicroRNA-665 facilitates cell proliferation and represses apoptosis through modulating Wnt5a/β-Catenin and Caspase-3 signaling pathways by targeting TRIM8 in LUSC
Source: Cancer Cell Int. 2021 Apr 15;21:215. doi: 10.1186/s12935-021-01913-z (PMC8051054; doi:10.1186/s12935-021-01913-z)

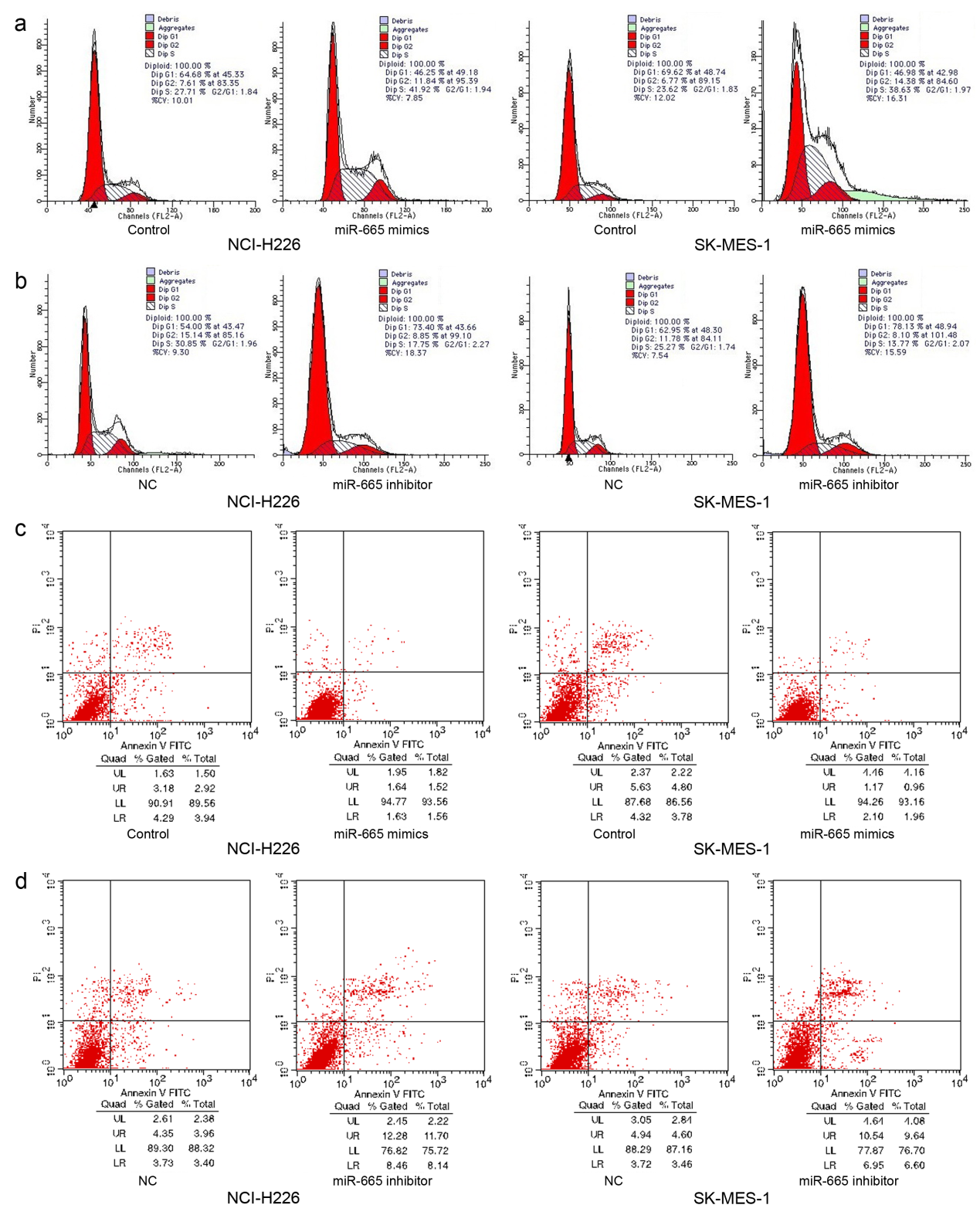


Figure S1


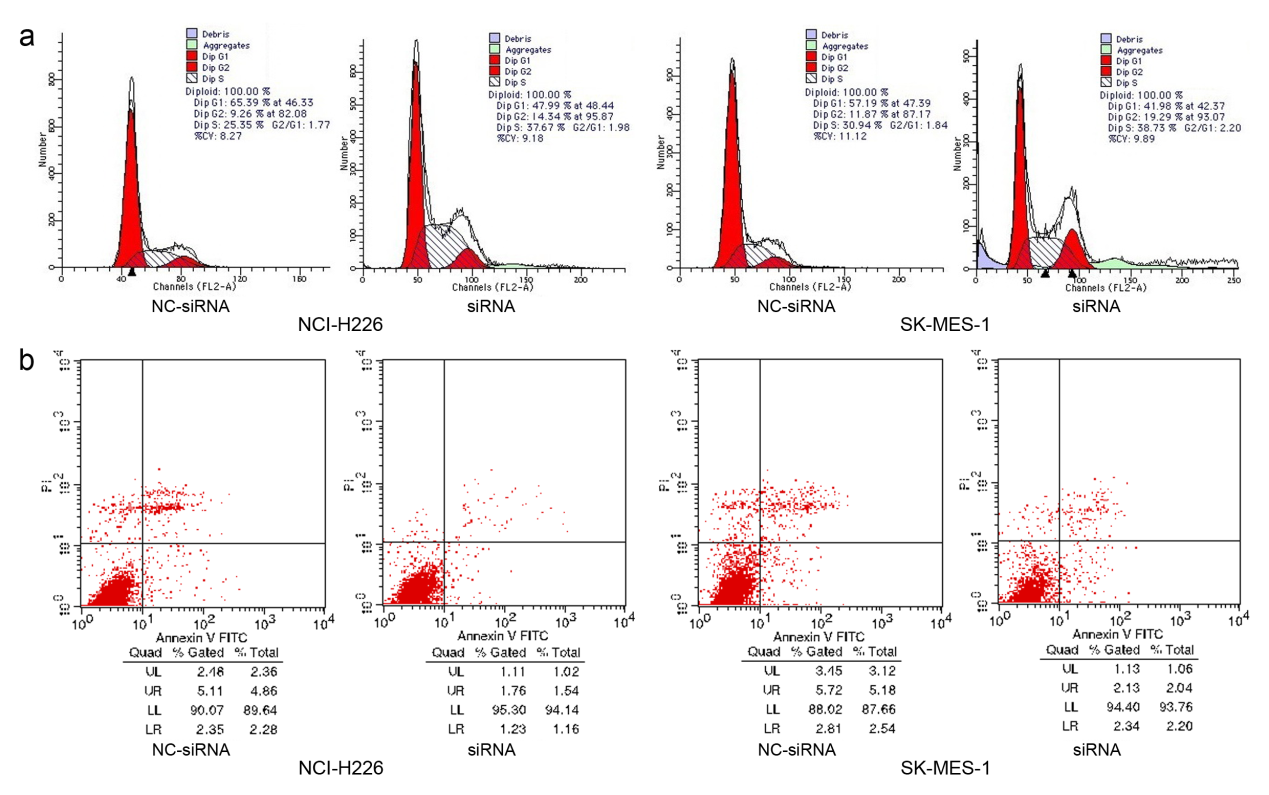


Figure S2


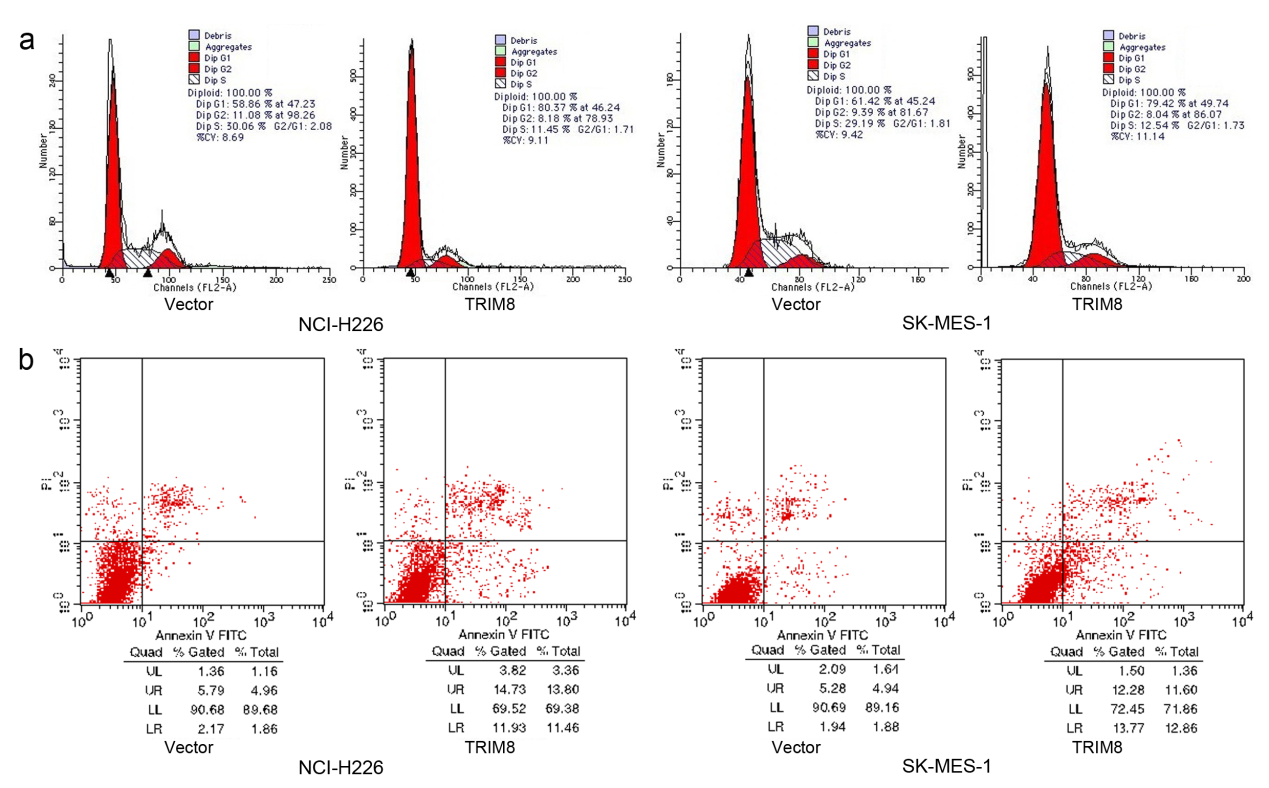


Figure S3

Supplement: Supplementary file 1 — Additional file 1: Fig. S1. The raw charts of flow cytometry. a Cell cycle was measured after transfection with the miR-665 mimics. b Cell cycle was detected after transfection with the miR-665 inhibitor. c Apoptosis was measured after transfection with the miR-665 mimics. d Apoptosis was examined after transfection with the miR-665 inhibitor. Fig. S2. The raw charts of flow cytometry. a Cell cycle was detected after TRIM8 siRNA transfection. b Apoptosis was measured after TRIM8 siRNA transfection. Fig. S3. The raw charts of flow cytometry. a Cell cycle was examined after TRIM8 overexpression vector transfection. b Apoptosis was measured after TRIM8 overexpression vector transfection. [file 12935_2021_1913_MOESM1_ESM.docx]
